# Exploring clinicians' perspectives on falls, balance and gait assessments to inform wearable device development. “Adding to the bigger picture of the patient in falls assessments”

**DOI:** 10.3389/fdgth.2025.1659786

**Published:** 2025-12-05

**Authors:** Henry Dunne, Clare Strongman, Sita Tarini Clark, Terry Fawden, Iwan Roberts, Manohar Bance

**Affiliations:** 1 Department of Clinical Neurosciences, Cambridge University, Cambridge, United Kingdom; 2 Vision and Eye Research Institute, Anglia Ruskin University, Cambridge, United Kingdom

**Keywords:** wearable, falls, gait, balance, qualitative, medical devices

## Abstract

**Introduction:**

Falls represent a major healthcare burden with significant costs to healthcare systems as well as individual patients. Falls assessments are complex, multifactorial and often involve input from multiple specialists. Collecting data to contribute to these assessments is typically done by clinical history and examination. Wearable monitoring systems that are capable of recording patient-specific data related to falls and falls risk factors may represent a rich source of information for patients and clinicians to inform their assessment and treatment plans. Here we present the findings of multimodal qualitative study that aimed to identify clinicians' perspectives on key features of falls assessments as well as the potential utilisation of wearable monitoring devices in the field of falls and falls assessments. Our findings inform device developers and ensure monitoring systems are designed with the end-users in mind.

**Methods:**

We used a multimodal qualitative study design. Survey responses from 26 healthcare practitioners (geriatricians, physiotherapists, falls prevention practitioners, and primary care physicians) were analysed using a reflexive thematic approach. Semi-structured interviews with practitioners were also conducted facilitating deeper analysis of the identified themes.

**Results and discussion:**

Six overarching themes were identified: “History of falls is key”, “Ecologically valid data”, Multidisciplinary approach, “Data that helps”, “Data causes anxiety”, and “Wearable literacy”. Exploration of these indicated that, if developed appropriately, wearable devices could support the clinical assessment and management of patients at risk of falls by providing ecologically valid, temporaneous and objective data related to modifiable risk factors of falls. This study outlines key priorities in falls assessment from clinicians' perspectives to inform device developers and highlight how a collaborative approach, engaging with clinicians, is necessary to facilitate development and integration of devices into patient pathways in a manner that is acceptable and useful to key stakeholders.

## Introduction

Falls are the leading cause of fatal and non-fatal injuries in the elderly population with one in four elderly people over the age of 65 falling at least once a year (1). As well as the physical and psychological costs that falls have on individual patients, they also have direct and indirect financial costs to healthcare systems. In the United Kingdom alone falls are estimated to cost the National Healthcare System (NHS) £2.3billion per year (2). Aging is also associated with several risk factors for falls including deterioration of gait and balance, cognitive decline, inactivity, and polypharmacy (3). As the global population continues to age, with in six individuals predicted to be over 60 years old by 2030 (4), the burden of falls will likely to continue to increase.

Given the numerous risk factors for falls, the clinical assessment of patients at risk of falling is complex (5). The National Institute for Clinical Excellence (NICE) advocates for multifactorial assessments and interventions to identify and modify any risk factors that contribute to falls. This includes patient-related risks, such as visual loss secondary to eye disease, or impaired mobility secondary to arthritis, as well as environmental risk factors such as home hazards or ill-fitting footwear (6).

Multiple risk assessment tools have been developed to support falls risk assessments; in fact, Strini et al. identified 38 different tools (7). These tools are typically numerical scoring systems with values assigned based on the burden of risk factors, determined by observation, clinical history and or examination findings. For example, STRATIFY is a tool, first validated in 1997 (8), used to determine falls risk in hospital inpatients, and scores patients' risk of falls based on the presence or absence of five risk factors. However, it's discrimination capacity is limited, with some studies reporting poor sensitivity and specificity of the tool (9). In fact, no individual falls risk assessment tool has demonstrated a sufficiently high positive predictive validity for differentiating those at high or low risk of falls (7, 10, 11). In an inpatient setting, NICE advises not to use falls risk prediction tools to predict risk of falling, but instead recommends considering all patients over 65 years old as at a risk of falls (6).

The British Geriatrics Society recommends completing a comprehensive geriatric assessment (CGA) for patients who presents with signs of frailty, including falls. This is an individualised multidisciplinary assessment encompasses a broad range of clinical tools to support the assessment (12). Implementation of a CGA in those at risk of falls has been shown to have a positive impact on patient frailty and falls outcomes in several randomised control trials (13). While accepted as a gold standard, a CGA is time and resource intensive assessment and is not always feasible. Barriers to effective CGA have been identified and include lack of partnership between the multidisciplinary teams (MDTs), logistical challenges in providing CGAs, and a poor acceptance of the benefits of preventative measures by patients (13).

A reliable falls risk prediction model would facilitate targeting of care to prevent falls and their downstream physical, psychological, and financial implications. A clinical tool with the potential to identiy modifiable risk factors of falls in individuals would also support targeted preventative interventions. A tool that monitored changes in modifiable risk factors over time would also support assessments of the impacts of an intervention, and highlight when further interventions maybe required to prevent an increase in falls risk. Technologies that might provide these tools are wearable devices.

Wearable devices are increasingly used across a diverse range of healthcare domains, facilitating the collection of patient specific data on various biophysiological parameters. The use of wearables is already established for continuous heart rate monitoring, blood pressure monitoring, electrocardiogram monitoring and blood glucose monitoring in relevant patient groups (14). There are numerous other physiological parameters that can be recorded and monitored over time via wearable technologies with applications across a full range of medical specialities. These include wearable devices which monitor skin health and hydration, to those capable of monitoring hormone and ion levels, to measurements of parameters of gait and balance and more (15). In Parkinson's disease five different wearable devices for gait analysis are conditionally recommended by the NICE to facilitate improved remote monitoring of movement features of the disease (16). Other wearable sensors have been investigated to enhance clinical assessments and rehabilitation (17) to estimate falls risk (18) and to identify near falls and falls (19). The question therefore arises, can these technologies can be leveraged to provide useful adjuncts to clinicians and patients within the domain of falls and falls management.

For a wearable device to be successfully integrated into clinical practice multiple barriers need to be overcome. Perhaps most pertinent, a device is redundant if patients or clinicians do not want to work with it (20). In turn, the importance of patient and public involvement and engagement in any health research is well established. Similarly, for wearables development, utilizing participatory design methods to ensure devices are acceptable to patients is recommended (21).

Several studies have shown that patient populations (including elderly populations) are interested in and happy to use wearable devices in various contexts and settings (22–26). However, equally importantly, any device also needs to be also acceptable to clinicians and healthcare workers whose practice will be influenced by the data it collects. Hence, involving relevant healthcare workers in the design process is also necessary. Few studies have been conducted to assess clinicians perspectives on the use of wearables in the field of falls, gait and balance disorders. The few studies that have tend to be device-specific and retrospective, that is, conducted after a device has been developed in order to gain device feedback, or involve only a very small numbers of clinicians (25, 27). However, prospectively engaging a representative number of clinicians before and during device development and implementation would represent participatory design and would help to ensure that a device would be useful and accepted by it's end-users.

Finally, another key challenge is the lack of understanding of the specific functions that a wearable monitor can fulfil in order to be most useful for patients and clinicians (28, 29). A comprehensive falls assessment and a CGA both utilise a broad and complex assessment involving the examination of multiple systems and is often performed by a variety of different healthcare practitioners. The two challenges above, namely involving healthcare practitioners in the design processes for a wearable for falls, and gaining and understanding of what specific uses healthcare practitioners would like a wearable to be able to fulfil, are critical to designing of a clinically useful and efficacious wearable in falls management.

Here we used a multimodal qualitative approach to establish clinicians' priorities in the assessment of patients with falls and to identify areas where clinicians find falls assessments challenging, in order to inform device development. We also aimed to identify clinicians' perceptions of the barriers to the successful integration of a wearable devices for falls in healthcare.

## Methods

Data collection consisted of a two-stage approach. Firstly, a qualitative survey with open ended questions was distributed to healthcare practitioners involved in the management of patients with falls—geriatricians, falls prevention instructors, primary care doctors (general practitoners) and physiotherapists. Initial responses were collected and analysed to identify key themes. A series of member reflections (one-to-one semi-structured interviews with participants from each of the four professions listed above) were subsequently conducted to support a deeper analysis of the identified themes (30).

The survey was first piloted by three clinical experts in the balance realm, as well as a qualitative researcher who was able to act as a critical friend (31, 32). This pilot highlighted a need to increase the openness of the questions to promote more detailed responses. The final survey is shown in Supplementary Material, and a summary of key questions is shown in Table 1 below.

**Table 1:** Summary of key questions included in the survey asked to the heathcare workers.

Number	Question
Q2	Describe typical falls assessments you conduct.
Q4	Outline any features that you think are crucial to undertaking a good falls assessment.
Q5	What are the possible outcomes of your falls assessments?
Q6	What do you think about falls risk assessment/stratification tools?
Q7	What is your experience of using wearable monitoring devices in clinical practice?
Q8	Do you think wearable monitoring devices could be useful in your job?
Q10	What are your thoughts on using wearable monitoring devices that collect data relating to patients gait and balance?
Q11	Do you/Would you have any concerns about using data collected from wearable patient monitoring devices?
Q13	What additional patient-specific data would you find most useful in your falls risk assessments? And why?

A purposive sampling method was the used to recruit participants with a diverse range of knowledge and experience managing patients with falls. Falls prevention instructors were identified after attending local in-person falls prevention exercise groups, during which instructors were given details of the study and invited to complete the survey. Geriatricians, and community geriatricians involved in local falls services, general practitioners, and physiotherapists were also approached either in-person or via email. Specific healthcare workers with interest in falls were chosen. Experience with using wearable devices or use of medical devices were not considered when selecting participants. All participants were provided with details of the aims of the study including “to investigate current practice in falls assessment and the possible utility of wearable monitoring systems in falls assessments.” Purposive sampling was used in order to ensure responses collected were from the “end-users”, the practitioners who manage patients will falls and balance disorders. This was done in order to obtain their expert opinions related the topic of falls assessments and wearable devices to provide device developers with their perspectives.

The survey responses were analysed using a reflexive thematic analysis approach (33). Researchers used immersive methods to code and recode the responses to support development of themes that comprehensively describe and identify meaning from the most salient features of the data set. An inductive approach using reflective practice was used to identify meaning from the data, emphasising the responders' views beyond semantic analysis and to uncover latent meaning within the responses, and the relationship between individual responses. In our reflective practice we recognised the importance of foregrounding the participants voices to limited the influence of the researchers' conscious and unconscious biases. Responses to surveys continued to be collected during the initial coding process. While no pre-determined number of responses was set to achieve thematic saturation, the decision to stop collecting responses was made at a point whereby our research question could be rigorously assessed based on the quality and volume of responses. Factors affecting this decision included ensuring at least three responses from each subset of health care workers, the overall assessed quality of each response, and the ability to identify new codes and themes from responses (akin to thematic saturation) (34). Having identified themes, mind mapping exercises and discussions between researchers were subsequently performed to further refine each of the themes.

The member reflections consisted of one-to-one semi-structured interviews with participants from each of the four professions listed. An interview guide was generated after critical review by researcher CS and after performing a pilot member reflection with a primary care doctor (Supplementary Additional File S1). Four semi-structured interviews were conducted by researcher HD via online video conferencing. Each interview was recorded and transcribed. The transcripts were then analysed using a reflexive thematic approach by HD and CS. Coding and findings from the member reflections were then compared with the themes identified in the survey responses. After further mind-mapping and discussions to refine themes, final themes were agreed.

The consolidated criteria for reporting qualitative research (COREQ) checklist was considered in the reporting of this study as was the BQQRG a value-based approach for reporting research (35–37).

### Positionality statement

Researcher positionality is intrinsically tied to the decisions made in the research, and in the interests of potentially reducing subjectivity and increasing generalisability in this study, the researchers' positionality and reflexivity is highlighted here to frame the interpretations and decisions made. Researchers had a mixed positionality which facilitated a pluralistic approach to our analysis (38). Our researchers consist of a Ear Nose and Throat surgical trainee with an interest in balance disorders, and a mixed methods researcher specialising in long term health conditions and the use of technology to identify gait changes, who also is a qualified postural stability instructor, and works within the local community delivering falls prevention classes as part of a multidisciplinary approach. Our researchers have frequent interactions with colleagues from a range of specialities including physiotherapy, primary care and geriatrics. We are not directly involved in the development of wearable technology for falls, however HD currently works in a research capacity one day per week in a lab alongside engineering colleagues that are seeking to use a wearable device to better understand gait in patients with balance disorders, and CS has a background in software engineering and has extensive experience of the development and validation of wearable devices.

## Analysis

Here we present the results and discussion together such that our analysis is integrated with contextual understanding and theoretical connections (33).

The questionnaire had 26 responders in total. Consisting of nine Geriatricians, three falls prevention health advisors, six primary care physicians, and eight physiotherapists.

Upon analysing the questionnaire responses, six primary themes were identified. These themes are illustrated in the Table 2 below.

**Table 2:** Displays each of the 6 identified themes, with various quotations from the survey responses which supported the identification of each theme.

Theme	Quotation
History of falls is key	“Take a history of any falls, what happened, prodromal symptoms…” GP5
	“Clinical history, particularly looking at past falls and to try to derive any features that may be indicative of syncope/seizures…” G1)
	“…we have a large proportion of confused/ cognitively impaired patients” P1
	“Any witness sightings where memory of the event is impaired can be vital.” GP2
Ecologically valid data	“The ability to monitor patient at home in day to day function and determine true risk” P3
	“Discuss how they manage mobilising around the house GP4”
	“…Individualised to the patient and their circumstances, consider their environment…” GP1
	“…Dynamic and re-assessed as conditions change” GP1
	“…are there tipping points or time points where falls are more frequent?” G5
	“Home visits are quite time limited so this would just be a basic assessment…” GP1
MDT approach	“a falls assessment is ideally a MDT approach but I will just cover the medical aspects of my falls assessment…” (G3)
	“In clinic where I am seeing a new faller, I would do it all myself” G1
	“…Multifactorial assessment noting as many risk factors for falling as possible-thinking about cardiac causes, medications, neurological causes, musculoskeletal causes, infective causes, environmental causes…” G4
Data that helps	“This could provide us with more data towards the reasons why they fall” FPI2
	“Observe gait” GP5 “Rhomberg’s test” G1
	“Early alerts. Monitoring progression/regression. Providing data on adherence to medications/exercise. Monitoring effects of intervention.” FP1.
	“I also would question usefulness of a wearable tool as a large proportion of patients have non modifiable causes for falls” P1
	“Ideally identifying all possible risk factors and then identifying modifiable ones” G2
	“Can also cause anxiety and be hard to interpret as we do not know how accurate this data is. We do use ambulatory home blood pressure monitoring and it is useful to see the trend over 24 hours. We also use 24 hour ECGs.”
	“Gait and balance data might be a good motivator for patients and a visual tool to demonstrate deficits or strengths”. FPI 3
	“Would need clear guidance on how to interpret and act on the results” GP1
Data causes anxiety	“I would need more information and training on normal ranges…” G1
	“Analysing patients who are acutely unwell may give false readings” G6
	“Can also cause anxiety and be hard to interpret as we do not know how accurate this data is” GP1
	“The patient might use the device incorrectly” FPI 1
	“…as long as protected by trust governance then no issues.” P8
Wearable literacy	“If patients are falling, you have probably already identified that they have issues with gait and balance.” G2
	“No experience at this moment in time, it could be useful.” FPI 2
	“I do not have any experience of using wearable monitoring devices” P1

Themes were then presented to clinicians during member reflection one-to-one discussions to provide greater insight into the significance of the themes. Illustrative quotations are highlighted in Table 3 below:

**Table 3:** Displays each of the 6 identified themes, with various quotations from the member reflections which support the development of each theme.

Theme	Quotation
History of falls is key	Sometimes you get very limited information, other times the history is very clear.
	I'd also be trying to sort of have a chat to the family as well, just to find out whether there is correlation because you can have a patient that tells you, oh, no, I don't fall at all type at all. And then you repeat their family, they fall like every few days.
	Regarding instances where it is not possible take a reliable history: that's a game where you think wearables. If you're if there's an unclear history, then wearables might be able to support getting in the future a better history.
Ecologically valid data	Home visits can be useful to look at environmental hazards.
	I don't think you'll ever be able to complete the whole assessment in a single place
MDT approach	I guess as physios we have a role… but I wouldn't say I'm a specialist within but is thinking about the vestibular system and is there an issue within the particular patients that are frequent fallers, are they hitting their head? Could there be a vestibular element to some of this as well?.
	We haven't got specific falls prevention teams that's made-up of all the right clinicians,…So you then have a 14 week delay in some cases, because that's how long it could take us to go and see the patient and then we identify that those things are for the GP to address and then we send it back…
Data that helps	If someone is having falls or if someone is having a symptom, it needs investigation and management and the more data you have the better. Obviously, if they come in with data that I can't understand, then I have to find someone who can…
	I mean at the moment all I do is if I suspect postural hypotension be the "cause. I just changed their medication. And then if it stops happening, then I don't do anything else, but I never actually know whether they're still at risk because they're still getting a degree of postural hypertension if they say they feel fine, I just accept that.”
	So I think that would be really interesting to understand a bit more, you know about the fall itself and and I guess just wearables have the technology to look at, you know your balance, your speed, your posture. Help maybe understand the fall itself in a different way.
	It could be quite motivational for patients as well to have an objective measure.
	you sometimes you do a time to get up and go and you just basically watch this. So it's a very observational. So there's not much, you know, standardisation potentially around it.
Data causes anxiety	…where that data being held…we need to make sure the data is being used appropriately
	Need to have robust safety mechanisms, but also not increasing anxiety in patients just looking at what the numbers are saying because actually it needs to be proportionate to the patient, because otherwise you're gonna get an influx with everyone being very worried that, their balance has dropped or their heart rate's gone very high. There could be a burden the other way, which is obviously not what we want.
	If you're the clinician that's actually saying, I want you to use this device, then you, you are ultimately responsible for making sure that you check that data and you act on it appropriately, yeah.
	It is a massive shift in technology and I think that probably will be one of the barriers—I think it would be that fear of of the unknown fear that of that you're going to have something that you don't quite know how you how to use.
Wearable literacy	“So I don't fully understand the range of things the device can measure”
	I mean the most common wearable monitors are those fitted by the cardiologist for monitoring ECG. There's, you know, you have to pick who you refer because there's so few of them available. The other is ambulatory blood pressure monitor monitoring. And obviously those devices are not very common. It's not very easy to do it. The so it's really access to those monitors. I'm unaware of any other monitors available…
	So with that that kind of interests me and then with a view to prevention but also intervention potentially if you've got a device that can actually influence if there's risky behaviour….

### Theme 1: History of falls is key

‘I'd also be trying to sort of have a chat to the family as well, just to find out whether there is correlation because you can have a patient that tells you, oh, no, I don't fall at all […] And then you [ask] their family, [and] they fall […] every few days.’

Consistently, across all specialities represented in our sample, healthcare practitioners emphasised the importance of the clinical history as the richest resource available in terms of identifying key contributing factors to a patient's fall. Conversely, it was also commonly expressed that acquiring an accurate history from a patient who has fallen is often not possible either due to either amnesia caused by the fall, or pre-exisiting cognitive impairment. The importance of a secondary source of information from a carer or witness of the fall was underlined as vital, though this was reported to not always be possible.

This dichotomy represents an area of interest for device developers—a wearable device's ability to identify a fall or near fall event, combined with its potential ability to collect accurate information about patients' movements, physiology, intentions and distractions, would provide context of the events leading up to a fall would be of interest to clinicians particularly when an accurate history is not attainable.

‘…if there's an unclear history, then wearables might be able to support getting a better history [in the future]’

It was suggested that a good history facilitates the identification of the cause of a fall. Taking this concept one step further, in member reflection 3 the geriatrician proposed that as well as differentiating the cause of a fall, one might be able to categorise and differentiate falls themselves… “…*hip fractures, then you have a lot of strokes for rehab. And do we need to think about falls in those populations slightly differently? are they different, could they getting a different types of fall?.*” Distinguishing types of fall as well as causes of falls could provide futher useful information both in terms of managing the acute effects of the fall, as well as developing patient-specific interventions and treatment. It is unlikely that a fall would be able to be definitively classified into different types based on history alone, however if fall and gait specific data is collected at the time of a fall this classification could be supported. Again this demonstates the clinical utility of having a device that capable of collecting data and linking it to a fall event. This could be useful to clinicians not just in identifying the cause of fall but potentially categorising the type of fall which may impact future treatments and management.

### Theme 2: Ecologically valid data

‘…the [home] environment plays a massive role in falls prevention’.

Most of the responders' experience of falls assessments take place in a clinical setting on hospital wards or their clinic rooms. However, the responses recognised that a good assessment of falls risk requires an understanding of the patient's home environment. This not only facilitates identification of potential environmental hazards which can be physically addressed (such as lack of handrails, a high step, or inadequate seating), but also facilitates an idea of how “*patients navigate their home environment*” and therefore a truer reflection of their “*real world*” gait and balance, reducing the Hawthorne effect/performance bias. The importance of this is reflected in the current clinical requirement for multidisciplinary input in the management of falls, including from community services.

‘Home visits are quite time limited so this would just be a basic assessment, and I would refer the patient to community services for a more detailed assessment if I was concerned’.

Moreover, performing multiple assessments over time was highlighted as important to assess impact of interventions and monitor patients' health and respone to interventions. Here some responders noted the utility of certain risk assessment tools for documenting changes in risk scores overtime. On the other hand, others reported risk assessment tools “*are too reductive*” and are often based on subjective measures which have limited utility.

It follows that a wearable device capturing objective data related to gait and balance in a patient's real environment and at multiple time points would offer healthcare practitioners with a tool that can provide information that they recognise to be important in a falls assessment, that is otherwise not readily available.

### Theme 3: Multidisciplinary approach

‘A falls assessment is ideally a MDT approach, I will just cover the medical aspects of my falls assessment'

A ubiquitous theme amongst responders that interlinked across all the themes and subthemes of the response analysis was a recognition of the role of multiple inputs during comprehensive falls assessments. This theme was not exclusively related to involving multiple specialists but also represents the fact that within a single assessment there is a need to examine multiple systems and perform a “*broad holistic assessment*”*.* Despite this, it was noted that multidisciplinary inputs are not always possible and sometimes “*I would do it all myself*”*.* Others report some elements of the assessment are required but currently there is not facilities or expertise available to complete them. For example: “*we currently do not complete a regular vestibular assessment, but we are in the process of creating a vestibular screening tool*”. Through member reflections we also came to understand that not only was MDT input not always accessible, it also frequently led to significant time delays for patients.

The benefits of the multidisciplinary and multisystem approach centres on it's ability to conduct comprehensive assessments of a patient to identify key contributing factors to their relative frailty and risk of falling. For a wearable device to be beneficial it should support these assessments by providing insights into these factors. Responders across each group of healthcare practitioners indicated that they often refer patients for: physiotherapy assessments and interventions, assessment of cardiovascular systems, assessments of vestibular systems and neurological assessment including identification of seizures as part of a multidisciplinary falls assessments. The function of these systems are potentially measurable by wearable devices; a wearable device for falls assessments should be capable of recording parameters related to these systems—in doing so it would support a multidisciplinary approach to falls, and could potentially reduce delays in completing such assessments by providing relevant data to specialists without additional waiting times.

### Theme 4: Data that helps

‘… the more data you have the better. Obviously, if they come in with data that I can’t understand, then I have to find someone who can…’

Wearable technology can generate lots of data about a range of physiological parameters, but which parameters would be most useful for patients and clinicians? We identified a subtheme of clinically modifiable data, as clinicians emphasized the importance of being able to take actions to improve a patients condition and thus their falls risk.—“*if its not clinically modifiable it is probably not useful*”. In the responses to our questionnaire the most commonly referred to “clinically modifiable data” related to falls was data of the cardiovascular system including ECGs and blood pressure monitoring. The relative prominence of monitoring cardiovascular risk factors in our responses may also reflect the fact this data is readily collectable, with well established pharmacological treatments are available, such that clinicians are familiar with utilising data. We also recognise the fact that our responders views will be biased by their personal expertise and practice as to what can and cannot be modified and so recommend thoughtful consideration before deeming a risk factor non-modifiable and therefore less useful.

Gait abnormalities may also be clinically modifiable (e.g., establishing treatment for Parkinson's, or commencing physiotherapy after a hip fracture). However, although respondees mentioned that a gait examination forms an important part of a falls assessment, objective assessments of gait were not frequently referred to. In member reflections clinicians highlighted the subjective nature of gait assessments:

‘my gait assessment is observe them walking 15 meters into my clinic room…’ ‘you sometimes you do a time to get up and go and you just basically watch this so it's a very observational. So there's not much, you know, standardisation potentially around it.’

Responses suggest objective data of gait parameters could support clinicians in: identifying pathology associated with typical gait forms, tracking trends over time which could be used to assess responses to treatments, using the objective data as a motivational factor in rehabilitation, and even potentially as an intervention itself by providing cues to improve gait when an issue has been identified. Other than one respondee mentioning gait speed during an assessment, we did not find reference to possible utility of identifying specific gait features as part of falls assessment.

The lack of emphasis on objective gait measurements in many of the responses maybe because data from these measurements is unfamiliar and not currently available to many healthcare practitioners. If a wearable device was developed to provide objective gait information related to falls, it would be crucial that the information it collected could be understood and utilised by the relevant healthcare practitioners accordingly.

### Theme 5: Data causes anxiety

…you're gonna get an influx with everyone being very worried that, you know, either their balance has dropped or their heart rate's gone very high…

It was acknowledged that collecting more information about a patient can also have negative implications for both the patient and the clinician involved in their care. Clinicians highlighted concerns regarding workload and responsibility around acting on results collected by a wearable. Designing a device that can collect useful data continuously or at multiple time points in a real-world environement about multiple systems including relating to the specialities of a MDT is supported by the themes we have previously outlined in our analysis. However, this also poses considerable practical issues. Who would be expected to act on the results of specialist data, particularly if the clinican with primary responsibility for the care of the patient may not have the time or expertise to do so? Equally, does frequent or continuous monitoring requiring frequent or continuous clinican review of the data? What safety measures would need to be put in place to support a patient if a significant abnormality was identified on day X and no one was available to interpret and act on the data until day Y? Safety measures should be built into any device that aims to support falls assessments, to alert both patients and clinicians if significant data has been collected. The threshold for signficiance would need to be carefully selected to avoid further anxiety as highlighted in our responses. This is necessary to both prevent overburdening the clinical team with large amounts of data to review as well as to avoid unduely worrying patients that abnormalities have been identified. As well as the risk and anxieties associated with false positive results, we also identified concerns regarding false negatives and the fact a device might prevent healthcare practicioners from identifying an issue or provide false reassurance. Device development should be done alongside relevant patients groups and clinical groups and should seek to recognise and address these anxieties.

Clinicians highlighted data security and privacy concerns was a key issue that would need to be addressed should a wearable device for falls be introduced into clinical practice. However, responders appeared to accept that any device would only be integrated into clinical practice if robust data protection and data governance systems were in place. In turn, this did not appear to be a cause of concern for clinicians. However, they did recognise patients may well hold these concerns and anxieties. Again these findings supports the fact that patient groups should also be included as part of participatory design processes, including in the development of governance frameworks and data security plans.

### Theme 6: Wearable literacy

‘Not as useful as ECG data, if patients are falling, you have probably already identified that they have issues with gait and balance.’

‘I do not have any experience of using wearable monitoring devices'

Responders were in general very open to utilising wearable monitoring devices to support falls assessment however the majority of responders had limited awareness or experience of using any wearable devices in healthcare currently. Other than well established at home cardiovascular monitoring systems (ambulatory blood pressure montitoring, and 24 h ECGs) most clinicians were not familiar with any other wearable devices that could be used to support falls assessments. There was some recognition of the utility of falls alarms, heart rate monotiring via smart watches, and three particpants referenced gait monitoring systems specific for Parkinson's disease patients.

Interestingly, when asked what data they would most like a wearable system to collect, clinicians offered a wide variety of suggestions that could support their falls assessments as highlighted in the Table 4 below.

**Table 4:** List of various data points and parameters that clinicians highlighted would be helpful to record to support their falls assessments.

Directly related to gait/activity	Other parameters
Recording number of falls	Blood glucose measurements
Recording timing of falls	Blood pressure and syncope measurements
Recording near fall events	Bone density data
Measurements of baseline mobility	Cardiac data (ECG, heart rate and rhythm)
Data on patterns of gait and mobility	EEG data during fall
Time spent sitting or lying per day	Signs of cognitive decline
Step count and cadence	Eye movement data
Muscle strength	Hearing assessments

Our analysis suggested a lack of “blue sky thinking” regarding data collected from wearable devices in patients with falls. Many of the responses disregarded the potential clinical significance of collecting data using a wearable, and instead referenced collecting data on cardiovascular systems e.g., ECGs and blood pressure as most useful to aid their assessment. This, in part may have been to the framing of our questionnaire and it not encouraging such thought processes. However, despite including the question “What additional patient-specific data would you find most useful in your falls risk assessments? And why?” responses tended to refer to data points that are already readily available or collectable. This may reflect entrenched thoughts by clinicians who may have a tendency to seek familiarity (e.g., preferring using results that can they are comfortable to interpreting and respond to). This points again to a subconscoious bias held by clinicians in response to the fact that some data causes anxiety. This again highlights the importance of device developers working alongside clinicians when designing wearables to be used in falls assessments but also the need for device developers to ensure clinicians are well informed the specific measurements a device might be able to collect in order to see how it could impact their practice and a patients outcomes. Moreover the fact that numerous responses reported little to no experience with the use of wearable devices in clinical practice emphazises the disconnect between emerging technologies and healthcare services and workers. Recognising limited experience and a lack of “wearable literacy”, much like the emerging need for training in healthcare related artificial intelligence (39) our findings support the need for education programmes to upskill healthcare workers in the use and design of medical devices and wearable technologies going forward.

### Overall

**Figure 1:**
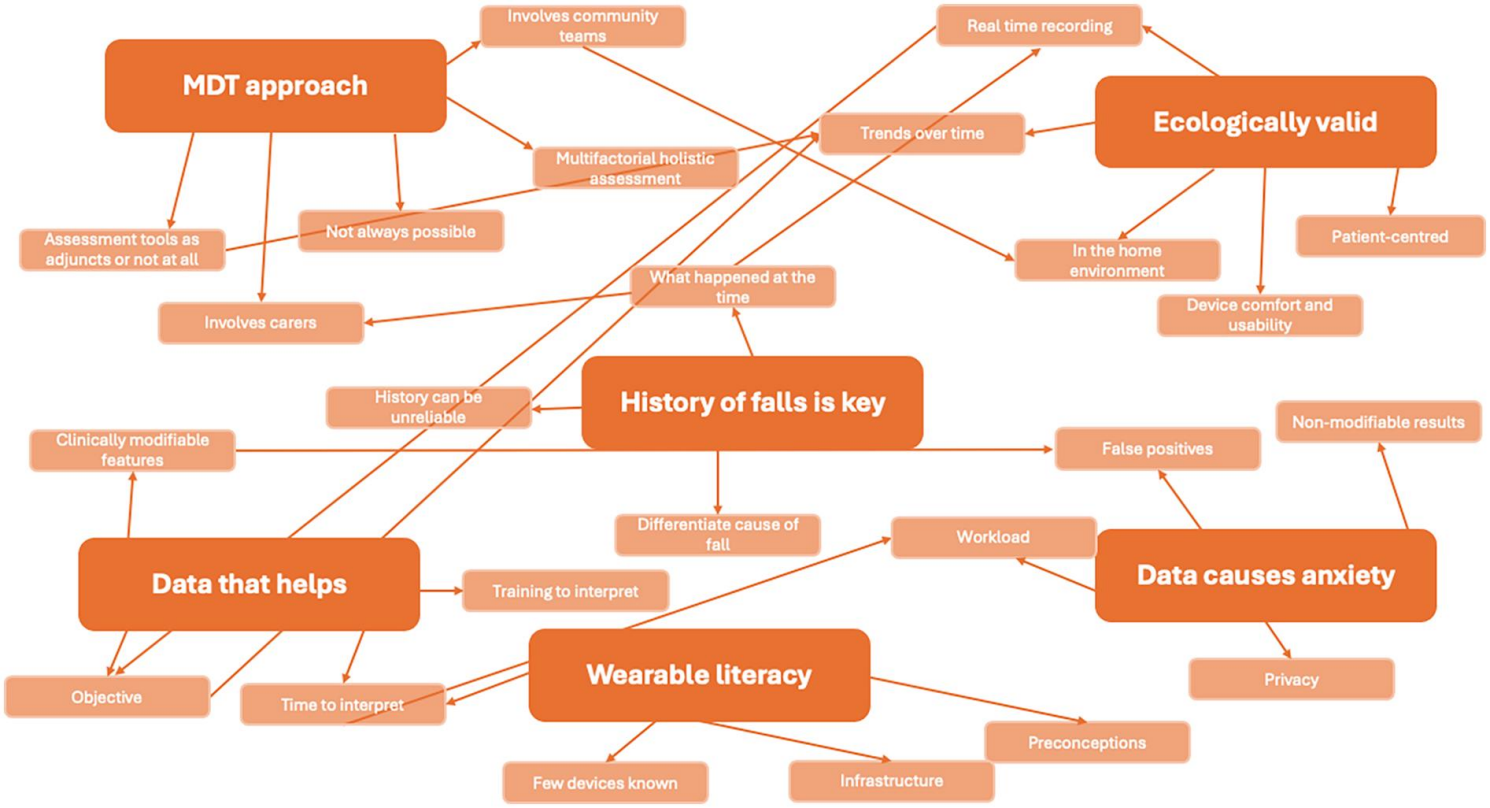
Representative mind Map: themes in red boxes represent overarching themes. Themes in other colours are subthemes link to overarching themes.

This qualitative analysis explored clinicians' priorities for falls assessments, their attitudes towards wearable monitoring devices and how such devices might be integrated into clinical practice to support falls assessments and management. Key themes and subthemes are presented in Figure 1.

Several studies have evaluated patients' perceptions of using wearables within domains linked to falls including gait and balance (22–26) and have highlighted an openness towards their utility, including within the elderly population (26). These findings align with the findings of our study, including the theme of “data that helps” and “ecologically valid data”, in which clinicians demonstrate an openness to wearables by emphasizing their potential usefulness. The experiences and opinions of academics and researchers working on developing devices to measure gait and activity have also been investigated (29). This study highlighted that wearables represent a potential paradigm shift in the way in which we can understand patients' behaviours and how they change in health conditions. These studies have also shown a disconnect between the researchers and the end-users (the patients and clinicians) (29) with a lack of joined-up-thinking and collaboration with patient groups and clinicians in the development of monitoring devices leading to challenges to successful integration of devices into routine clinical practice. While a paradigm shift has been purported, our study finds several hurdles to be overcome before such a shift may take place from the perspective of the healthcare workers. Lack of wearable literacy, and a number of anxieties relating to data from wearable devices suggest that a integration of wearable devices into healthcare in a slow and steady fashion is more likely than any paradigm shift. Few studies have attempted to investigate clinicians' perspectives of wearable monitoring devices within the domain of falls (25, 27) and typically these represent evaluations of a device that has already been developed.

As the end-user with ultimate responsibility over the data collected, the clinicians' perspectives must be considered throughout all stages of device design and integration into clinical practice. This scoping study sought to improve researchers' understanding of clinicians' perspectives regarding falls assessments to inform development of devices within this domain.

Our findings reiterate the importance of a comprehensive geriatric assessment for the assessment of a patient at risk of falling. Clinicians recognise the importance of a detailed history of a fall to ascertain causative and modifiable risk factors for falls. They underlined the importance of a multidisciplinary approach which reflects the fact risk factors for falls relate to multiple systems spanning musculoskeletal health, cognitive health, cardiovascular health, vestibular function, psychological health, and social situation, each of which may require specialist input. The importance of understanding health in the context of each patient's real-world environment was also emphasized.

Challenges to satisfactorily achieving a CGA were noted and represent an important insight for device developers. A device that collects temporaneous data around the time of a fall could provide vital insight for clinicians who are limited in their ability to take a reliable history due to patient factors such as amnesia or cognitive decline. A device that collects objective data relating to any of the multiple modifiable risk factors to falls might facilitate a clinician initiating management of a patient and reduce delays linked to waiting for specialist input. Collecting real-world data in real-time relating to patients' mobility and gait would also provide greater insight into their level of functionality supporting holistic, patient-centred care and interventions. This would facilitate longitudinal data collection to predict worsening of a patients clinical condition.

This study identified that clinicians' views on what represents a modifiable risk factor and or a data point of interest may be biased by their own experiences and areas of expertise. Device developers should bear this in mind when collaborating with clinical partners. The responses also suggested that where subjective measures are commonly used in clinical practice, for instance in the assessment of gait, the potential significance and utility of more objective data was not always appreciated by clinicians. The identified responses fell into one of two groups, namely, those who felt subjective assessments were adequate and therefore did not require any objective measures, and those who felt objective measurements would improve their assessments. Device developers should consider ways in which they present objective measurements of gait to ensure clinicians in both camps to maximise benefit. Some of the responses (more frequently amongst the physiotherapists) in this study referred to utilising validated clinical scales relating to balance and gait when completing falls assessments including the Berg Balance Tests and Timed Up and Go assessments; presenting data on gait and balance in a format previously known to clinicians such as one of these could be a useful approach (40, 41).

We found in general an openness to use of wearables to support falls assessments amongst clinicians—with many suggesting specific cases in which a wearable device could be useful. These included, obtaining data about cardiovascular system at time of a fall, as well as patients general day-to-day mobility. Clinicians reflected on utility of wearables not only in terms of identifying risk factors for falls but also to as guiding rehabilitation efforts and as a motivational tool for patients. Despite openness to wearables, clinicians rarely suggested any advanced use-cases for such devices. Rather, their suggestions tended to be achievable with existing technologies currently perhaps because of a subconscious resistance to think outside of their current practice. Those collaborating with clinicians in the device design should ensure the healthcare practitioners are encouraged to explore the numerous exciting developments and achievements within the wearable field.

To alleviate concerns regarding what to do with, and how to act on the data collected by a wearable device we found that clinicians would expect clear guidance on how to interpret results. In many cases training may be required particularly if data collected encompassed multiple facets of an MDT's expertise. Describing reference ranges for normal data values when developing monitoring devices would support clinican learning and utility of any device.

We recognise that there are limitations to our data set and analysis. Although our purposive sampling methodology ensured that a range of perspectives from falls experts were collected, all responses were identified from healthcare workers working from within the same East Anglia region in the United Kingdom, which may limit the generalisability of our findings. There is also a risk of responder bias, those with interested in technology and wearable devices may have been more likely to engage in our study. As a qualitiative study we sought to provide and interpret true meaning from responses that is applicable to medical device designers and clinicians alike. We recognise it is not feasible to identifiy a universal and statistically generalisable truth by taking the reflexive thematic approach (42).

## Conclusions

Our analysis has facilitated a deep and detailed interpretation of responses uncovering semantic and latent meaning in responses to support the findings reported above. The reflective approach used in this study ensured that investigator biases and contexts were considered throughout the research and decision-making processes. Falls and assessment of falls represents an important challenge in healthcare today. Clinicians have described the complex nature of their assessments which are not always perfect due to system and patient factors. We have highlighted key parameters of a falls assessment and identified areas where data collected by a wearable monitoring device could be useful for clinicians. Clinicians described how collecting ecologically valid, temporaneous data associated with modifiable risk factors of falls linked to various health and mobility parameters could enhance their assessments. The way in which this data is presented to clinicians must be carefully considered to avoid generating unnecessary work and to ensure utility. A collaborative approach to device development that engages with relevant patient populations and clinical teams is necessary from initiation of device design to its integration into a patient pathway.

## Data Availability

The raw data supporting the conclusions of this article will be made available by the authors, without undue reservation.
