# Supplementary material for: Exploring clinicians' perspectives on falls, balance and gait assessments to inform wearable device development. “Adding to the bigger picture of the patient in falls assessments”
Source: Front Digit Health. 2025 Dec 5;7:1659786. doi: 10.3389/fdgth.2025.1659786 (PMC12715005; doi:10.3389/fdgth.2025.1659786)
Supplement: Supplementary file 1 [file Datasheet1.pdf]

Questionnaire table form

How often do you make falls risk assessments?

Why do you do falls assessment at the above frequency?

Describe typical falls assessments you conduct.

Outline any features that you think are crucial to undertaking a good falls assessment.

What are the possible outcomes of your falls assessments?

What do you think about falls risk assessment/stratification tools?

What is your experience of using wearable monitoring devices in clinical practice?

Do you think wearable monitoring devices could be useful in your job?

How do you think wearable monitoring devices could be useful in your job?

What are your thoughts on using wearable monitoring devices that collect data relating to patients gait and balance?

Do you/Would you have any concerns about using data collected from wearable patient monitoring devices?

Are you aware of any wearable devices utilised in falls detection or falls risk assessment? (If Yes please specify)

What additional patient-specific data would you find most useful in your falls risk assessments? And why?

Are there specific patient groups that you would like more data relating to falls, balance and gait? Why is this?
